# Challenges in Coding Adverse Events in Clinical Trials: A Systematic Review

**DOI:** 10.1371/journal.pone.0041174

**Published:** 2012-07-20

**Authors:** Jeppe Bennekou Schroll, Emma Maund, Peter C. Gøtzsche

**Affiliations:** Nordic Cochrane Centre, Rigshospitalet, Copenhagen, Denmark; Nottingham University, United Kingdom

## Abstract

**Background:**

Misclassification of adverse events in clinical trials can sometimes have serious consequences. Therefore, each of the many steps involved, from a patient's adverse experience to presentation in tables in publications, should be as standardised as possible, minimising the scope for interpretation. Adverse events are categorised by a predefined dictionary, e.g. MedDRA, which is updated biannually with many new categories. The objective of this paper is to study interobserver variation and other challenges of coding.

**Methods:**

Systematic review using PRISMA. We searched PubMed, EMBASE and The Cochrane Library. All studies were screened for eligibility by two authors.

**Results:**

Our search returned 520 unique studies of which 12 were included. Only one study investigated interobserver variation. It reported that 12% of the codes were evaluated differently by two coders. Independent physicians found that 8% of all the codes deviated from the original description. Other studies found that product summaries could be greatly affected by the choice of dictionary. With the introduction of MedDRA, it seems to have become harder to identify adverse events statistically because each code is divided in subgroups. To account for this, lumping techniques have been developed but are rarely used, and guidance on when to use them is vague. An additional challenge is that adverse events are censored if they already occurred in the run-in period of a trial. As there are more than 26 ways of determining whether an event has already occurred, this can lead to bias, particularly because data analysis is rarely performed blindly.

**Conclusion:**

There is a lack of evidence that coding of adverse events is a reliable, unbiased and reproducible process. The increase in categories has made detecting adverse events harder, potentially compromising safety. It is crucial that readers of medical publications are aware of these challenges. Comprehensive interobserver studies are needed.

## Introduction

The decision to prescribe a drug is based on the balance between the drug's benefits and harms. All drugs have unwanted effects and reliable information about these effects is important. Throughout a clinical trial, adverse events, including harms of the drug, are monitored and recorded for the purposes of patient safety, regulatory requirements, and developing a safety profile of the drug. The process of condensing thousands of pages of data on adverse events from clinical trials to tables in regulatory submissions and summaries in papers and product labeling is complex and involves many assumptions and choices. Readers of medical journals need to be aware of these issues in order to appraise published study reports critically.

Before harms are reported (or not reported) in a published paper, many decisions have been made. A patient in a trial may experience ‘something’. In some studies, patients can contact investigators by phone; in other studies, the symptoms may not be recorded before the next visit (which might be weeks ahead). The patient may or may not describe the experience to the investigator, partly dependent on the method of elicitation used by the investigator (e.g. open ended questions, symptom checklists). Information about adverse events can also be gathered from medical records and laboratory values. The investigator interprets the information in a biomedical framework and might filter some of it, especially if he believes the event is not drug related [1]. If the investigator decides to record the event, he will do so in the patient's case report form (CRF). This information will later be transformed by a medical coder employed by the trial sponsor. Coders use a medical dictionary, which is a predefined list of possible adverse events organized in a hierarchy, to code the narrative description of an adverse event [2].

**Figure 1:**
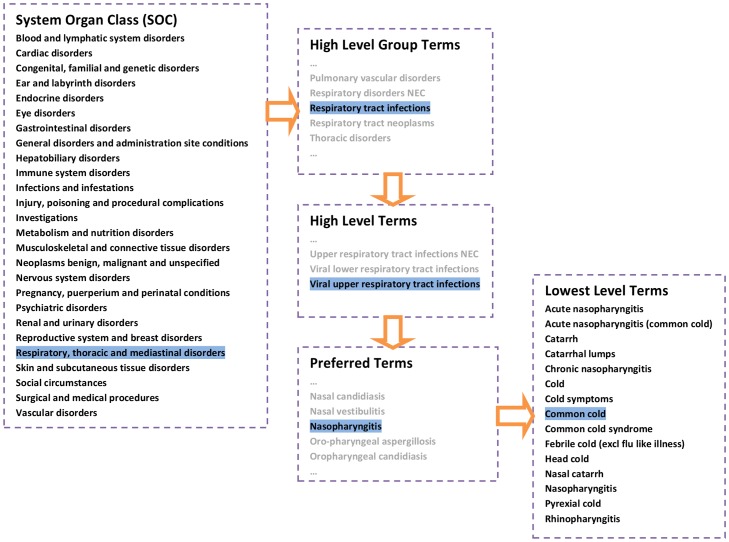
The MedDRA 5-level hierarchy demonstrated by using ‘common cold’ as an example.

Pharmaceutical companies have historically used many different dictionaries, such as WHO's Adverse Reaction Terminology (WHO-ART), the Thesaurus of Adverse Reaction Terms (COSTART), or the International Classification of Diseases (ICD 9 and ICD 10), to categorize adverse events, frequently customizing a dictionary for a specific trial. In 1994, the pharmaceutical industry, together with regulatory agencies, developed a standard dictionary named the Medical Dictionary for Regulatory Activities (MedDRA). Initially, the purpose was to allow standardized electronic submissions [3]. MedDRA is a five level hierarchy with Lowest Level Terms at the bottom, followed by Preferred Terms, and with System Organ Class (SOC) at the top (Figure 1). Events are initially coded with Lowest level terms and they consist of thousands of synonyms and alternative spelling of Preferred Terms. Preferred Terms are unique medical entities. Companies are not allowed to add new terms but can suggest new terms – or alternate placing in the hierarchy – which will then be considered for the biannual update. To ensure an adverse event is only counted once in the standard summary tables, each Preferred Term can have only one primary SOC but several secondary ones to aid data retrieval [3]. It is mandatory for pharmaceutical companies to use MedDRA when applying for approval in the EU and Japan. In the US it is the terminology of choice [4].

With MedDRA, each adverse event can be coded as several different terms, ‘insomnia’ could for instance be coded as 11 different preferred terms [2]. This may lead to inconsistency and failure in identifying harms [2]. At the end of the trial, data are categorized and summarized, and adverse events are lumped into broad categories for practical reasons. At each of these steps, decisions are made that might impact the overall impression of harms and might lead to important harms being missed, e.g. “gastrointestinal events” may include cases of nausea as well as bleeding ulcers.

Mislabeling of adverse events can skew the interpretation of a drug's harms. The antidepressant paroxetine was tested in adolescents in an infamous trial that initially declared that the drug was “generally well tolerated” [5]. The paroxetine group, however, had an overrepresentation of “emotional lability”. After scrutiny by the FDA and independent experts, it turned out that this term was only used when patients had “suicidal tendencies”. Other cases of suicidal tendencies had been coded as aggression or “exacerbation of depression” [6].

With paroxetine, the miscoding appeared to be deliberately misleading, but it illustrates some fundamental problems with coding. Small deviations from the ideal of objective coding can lead to significantly changed conclusions and are usually impossible for the reader to discover. Development of new drugs that make a difference to old drugs in terms of benefit is increasingly difficult, and many new drugs are therefore being marketed as having less harms than their predecessor. Hence, readers of the scientific literature should be particularly focused on harms, and whether they have been reported reliably.

Our objective was to conduct a systematic review of studies on intra- and interobserver variation and other potential problems related to interpretation and translation of adverse events (as reported by clinicians) into coding terms for use in clinical study reports (for regulatory approval) and in publications (for marketing).

## Methods

We searched PubMed, The Cochrane Library (CENTRAL and Methods) and EMBASE on the 28^th^ of October 2011 and updated the search the 9^th^ of March 2012. The search string was a combination of synonyms of adverse events and interobserver studies (see details in Text S1). Search terms also included the names of common dictionaries used for medical coding. We had no language or other restrictions for the searches. We also went through the reference lists of the included studies, visited medical dictionary websites and contacted principal authors for information about additional studies. Our protocol is available on request.

All abstracts and titles were screened for inclusion by two independent observers (EM, JBS) . Any differences were resolved by discussion. When eligibility could not be determined based on title and abstract alone, the full text article was retrieved. Eligible studies were interobserver studies of coding in clinical trials. Other studies addressing challenges in coding of adverse events in clinical trials were also included. Review articles were excluded.

We adhered to the PRISMA guidelines for reporting systematic reviews [7], see Checklist S1 for details. Because of expected heterogeneity in the results, our review was planned to be qualitative.

## Results

**Figure 2:**
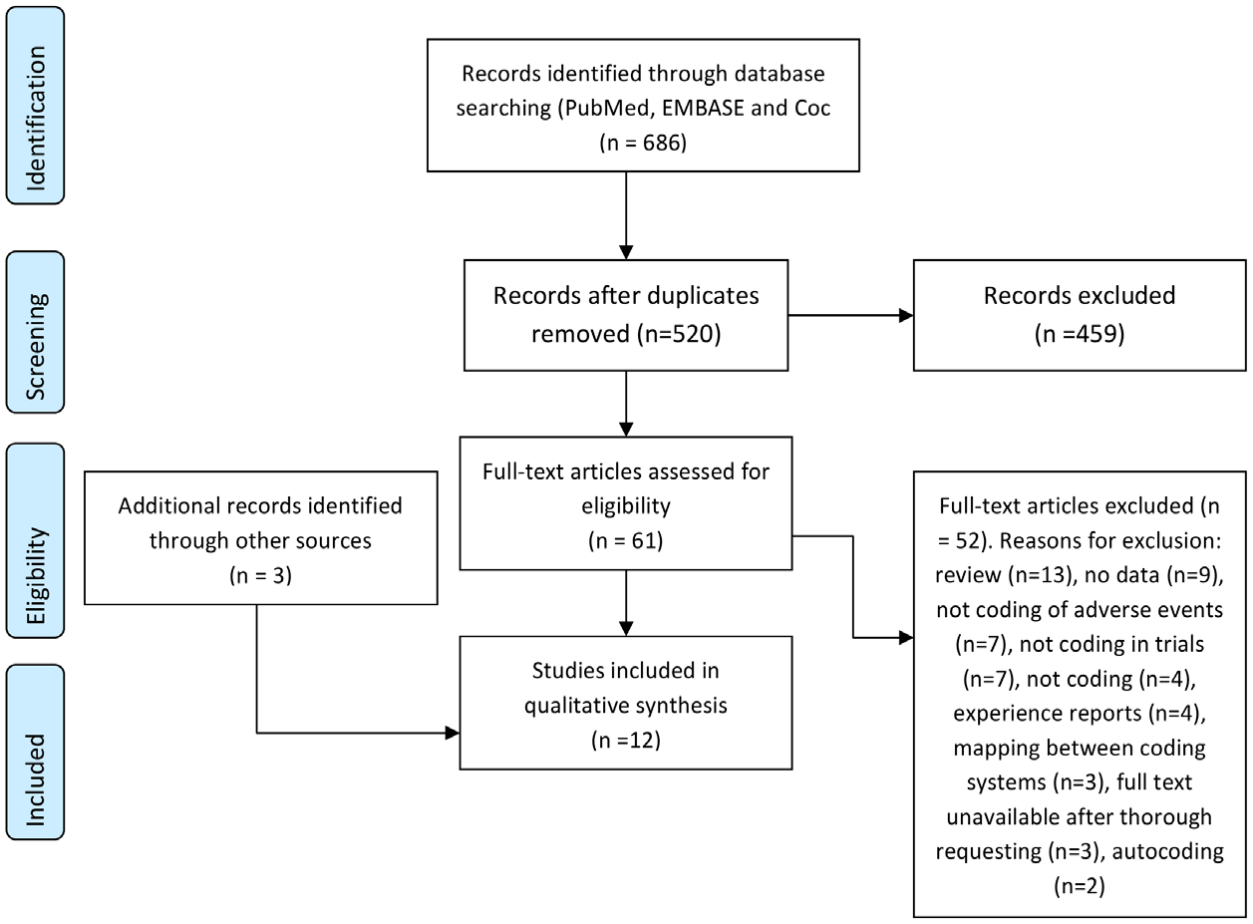
Flow chart of the process of identifying studies.

Our search returned 520 unique citations. We retrieved the full text for 61 articles and included 9 of these. The papers we excluded were reviews (n = 13), papers with no data (n = 9), not describing adverse events (n = 7) and papers not referring to clinical trials (n = 7) or otherwise not relevant (n = 16). See figure 2 for details. Three additional papers were included from the references of located papers. Only one of the included papers was an interobserver study of coding. All included papers are described in table 1.

**Table 1:** Description of included studies.

Author/Year	Aim	Study design	Main findings
Brown 1996 [9]	To determine MedDRA's adequacy in representing medical terms used in UK data sheets	A product from each of the main drug classes in the British National Formulary was scrutinised for medical terms which were then coded using MedDRA. Matches were classed for accuracy	Identical or acceptable matches for 90% of the side effects
Brown 1997 [10]	To compare MedDRA to the COSTART for specificity of coding clinical trial data and for the effects of coding on the analysis and presentation of safety data from the trial	Verbatim descriptions of adverse events from a phase II trial were coded by MedDRA and COSTART and the association was assessed for accuracy. The incidence of adverse events using the different dictionaries was compared.	Using MedDRA resulted in more exact matches than using COSTART (90% vs 62%). With MedDRA 267 codes were used, with COSTART only 169. The two terminologies gave different breakdowns of adverse events
Brown 2002 [20]	To explore the numerical and conceptual relationships between WHO-ART and the MedDRA and their ability to detect signals	A sample of approximately one sixth of all WHO-ART preferred terms was taken. MedDRA was searched for each of these terms to find the best match	315 WHO-ART terms were identified and were matched with 943 MedDRA preferred terms
Brown 2004 [2]	To identify common adverse events in clinical trials by looking at product labeling and comparing this to MedDRA terms	Adverse events from 10 randomly selected drugs in the Physician's Desk Reference were compared with MedDRA terms	Some terms in the product labels were associated with hundreds of MedDRA terms. E.g. “infection” (several hundreds) and “pain” (168 items)
Fescharek 2004 [21]	To investigate MedDRA's impact on retrievel strategies, analysis and presentation of coded data	Comparison of trial data coded in WHO-ART with the same data recoded in MedDRA	In WHO-ART 214 different terms were used; whereas in MedDRA 312 different terms were used. They were grouped quite differently
Journot 2008 [12]	To be able to use the MedDRA hierarchy for data analysis by redefining the hierarchy to fit trial objectives	The authors developed a new general 5-step strategy to select a SOC (system organ class) for an adverse event as trial primary SOC, consistent with trial-specific objectives. This was applied to clinical trial data and compared to the original MedDRA hierarchy	Altogether, 23% of MedDRA primary SOCs were modified
Nilsson 2001 [14]	To analyse the impact of defining “treatment emergent adverse events”	Since only treatment emergent adverse events are reported in trials the authors identified in how many ways this could be defined and the consequences on test data	At least 26 different strategies for censoring adverse events exist. Depending on the chosen strategy the same data resulted in 2 to 7 adverse events.
Toneatti 2005 [8]	To assess the feasibility of coding with MedDRA. To develop an approach for MedDRA implementation within an institutional research unit that contributes to an efficient, concise and reproducible event coding	1) Two blinded coders used MedDRA to code 260 verbatim descriptions of adverse events from a clinical trial and reported difficulties in coding. Variability between the two coders was measured and accuracy was determined by a medical coding committee.2) MedDRA 6.1 was applied to both the list of frequent adverse events and a trial coded with MedDRA 5.0	1) 32 adverse events (12%) were coded differently by the two coders; 13% of the adverse events were assessed to be “non-accurate”. 2) When changing to a new MedDRA version, 38 (9%) adverse events changed.
White 1998 [11]	To obtain a preliminary assessment of the impact of MedDRA on the frequency of expedited adverse event reports based on current (non-MEDDRA) labeling	Verbatim adverse event reports (surveillance) for two different marketed drugs were coded with WHO-ART and MEDDRA and it was determined whether the code was mentioned in the product label. A rating scale was used to quantify the differences	Twenty-seven terms (13%) had some syntactic differences although these were not considered medically significant. Thirty-two terms (16%) were rated as medically significantly different but did not affect the label. Ten terms (5%) were rated as both medically different and resulted in a labeling discrepancy
Zhao-Wong 2006 [17]	The purpose was to obtain more user input on issues related to the feasibility study and MedDRA terminology in general	A survey of MedDRA users performed by the MSSO, the organization maintaining MedDRA	Received 12 responses out of 29 invited. The majority of MedDRA users relied on primary paths for both re-porting and analysis. The usage of secondary links was limited
MedDRA Term Selection 2011 [13]	To aid medical coders in choosing codes consistently	Not a study but a manual	Describes many situations where there might be doubt on how to code a reported adverse event and suggests a solution
MedDRA Data Retrieval 2011 [16]	To aid investigators in presenting adverse events	Not a study but a manual	Describes how adverse events can be presented by the hierarchy and how to use standard and custom searches to lump related adverse events together

The only interobserver study of coding was done by Toneatti et al. who performed a pilot project where two experienced coders used MedDRA for the first time. They coded 260 events independently and a medical committee later determined whether the coding was accurate [8]. In 12% of the cases, the coding resulted in two different Preferred Terms. When the comparison was made higher up in the MedDRA hierarchy, the difference was smaller, around 5%, indicating that the different Preferred Terms were related to some extent. The 12% difference can, however, be important because statistical analysis of adverse events is often done at this level in the hierarchy. The medical committee determined that in 8% of the cases, which the coders perceived as easy to code, the coding was nevertheless inaccurate. The study was extended and 1640 events were coded. The inaccuracy rate was around 10% in the larger sample but interobserver variation was not reported.

A study by Brown et al. from 1996, the early days of MedDRA, found that from existing product labeling 90% of the terms could be exact or acceptably matched in MedDRA [9]. The next year the Brown et al. compared how accurate adverse events from clinical trials could be coded in MedDRA versus COSTART. This study also found that 90% of the matches were exact or acceptable with MedDRA but only 62% with COSTART [10]. The authors pointed out that the entire COSTART dictionary was imported in MedDRA.

White et al. looked at 204 post marketing surveillance events [11]. When the same verbatim text was coded with MedDRA and WHO-ART 32 pairs (16%) were rated as medically different. In 13 cases, the WHO-ART code was included in the product label and the MedDRA code was not.

In a paper from 2002 Brown was concerned about the increasing amount of terms in MedDRA. He showed that 315 WHO-ART terms could be mapped to 943 MedDRA terms. In 2004 Brown compared adverse events reported in the Physician's Desk Reference from 10 randomly selected drugs with corresponding MedDRA terms. He found that some adverse events (e.g. infection and pain) corresponded to hundreds of terms in MedDRA [2].

The constant updating of MedDRA has also been a source of concern. Toneatti et al. also examined the impact of updating from version 5.0 to 6.1. Out of 436 unique Lowest Level Terms, 38 (9%) changed either the Preferred Term or the SOC related to them, or both [8].

Each Preferred Term is associated with one primary SOC. This SOC is predefined by MedDRA and users are not allowed to change this or anything else in the MedDRA hierarchy. The most appropriate primary SOC for an adverse event might, however, differ from study to study. In an HIV trial, 23% of primary SOCs were altered when using a predetermined strategy to choose the most appropriate primary SOC [12]. It demonstrates the subjectivity of the hierarchy.

There is often doubt about how an adverse event should be coded and therefore it is necessary to develop “coding guidelines” for each trial. A 45-page manual has been developed by an expert group to address more general issues, which means that coding can no longer be performed by a physician without special training . If a diagnosis and several symptoms – that are included in the diagnosis – are reported, several strategies can be used in coding this data. One strategy is to code both symptoms and diagnosis, another is to code the diagnosis and leave out the symptoms that are included in this diagnosis. It is recommended that coders do not make diagnoses based on reported symptoms.

The manual offers specific guidance on how to handle suicide and self harm. It explicitly states that an intentional overdose should be coded as an overdose, and not as a suicide attempt. “Cut her own wrist” should be coded as “self inflicted laceration” and only as a suicide attempt if the verbatim clearly states that the purpose was suicide. The unfortunate consequence of these recommendations is that suicide attempts become much harder to detect in pharmaceutical trials.

Infections can either be coded by the microorganism or the anatomic location of infection. The current recommendation is that chlamydial respiratory infection should be coded as “Chlamydial infection” [13]. “Chlamydial infection” will then represent respiratory and urogenital infections, even though it is clinically relevant to distinguish between these illnesses. When creating a rigid system that exclusively categorise events, it will always be possible to find examples that, in a given context, should have been categorized differently.

Another important factor that will effect whether an event is coded or not, is the definition of “treatment emergent adverse event”. It is usually defined as any *new* adverse event or worsening of an existing condition after initiation of therapy [14]. Even though the definition seems quite clear, Nilsson et al. identified 26 different ways of defining treatment emergent adverse event. Depending on the selected strategy, the authors' test data returned from 2 to 7 adverse events [14]. One of the reasons for the many definitions is determination of initial severity. If the patient had several appointments before they actually got the active drug (run-in period), and they reported ‘headache’ but with varying severity during these visits it is unclear which severity should be used. It is very important because all following headaches in the actual trial with the same severity would not be considered an adverse event and would therefore not be coded [14]. The most important factor that influences the number of adverse events is the way that adverse events are compared. If a patient had “headache” at visit 1 and “head pain” at visit 2, does that represent the same event or are they different? Obviously, more details would be preferred but the two terms would normally be coded and then compared on a predefined level in the hierarchy of MedDRA. It will obviously make a big difference whether you compare verbatim text, Lowest Level Term or Preferred Term. If verbatim text is chosen, then “headache” and “head pain” would be considered two different adverse events [14]. Comparing on Preferred Term level would probably mean that the events were considered identical. The drawback is that you might overlook two medically different events that are lumped together. Obviously, more adverse events will be censored if the Preferred Term level is used. Brown et al. (1997) also looked at the impact of coding on the way harms were presented. Several new adverse events were detected using MedDRA because of splitting of existing groups. This meant that the list of the 10 most common adverse events changed substantially when MedDRA was used. They concluded that “use of a different terminology can alter the apparent safety profile of a drug” [10]. The same conclusion was reached by Fascharek et al. after coding the same trial data in WHO-ART and MedDRA .

In a survey of only 12 MedDRA users it was established that the usage of “secondary links” is limited [15]. “Secondary links” are searches and secondary SOCs that will make it possible to lump related adverse events together thereby increasing statistical power. MedDRA has more than 18,000 Preferred Terms and, as we have described above, there is a risk of signal dilution compared to previous dictionaries with less terms. Even the developers of MedDRA acknowledges that the hierarchy cannot be relied on to retrieve exhausting information about adverse events [16]. Several authors have prompted for regulatory guidance on MedDRA implementation [17]. The expert group states that simple summaries might not always be sufficient, and that you may have to explore the safety data in greater detail [16].

## Discussion

The only interobserver study of adverse event coding we found showed that 12% of the adverse events at Preferred Term level were coded differently by two coders. This could be quite significant for some trials but obviously it depends on what symptoms were coded differently and how. Important interpretation is done by the medical coder, and 8% of the coding was declared as medically inaccurate when rated by experts. This study has not led to further investigations of the subject, which is surprising.

The constant development of more terms in MedDRA might intuitively lead to less interobserver variation because there will be more exact matches to the verbatim text. Conversely, it might also lead to increased variation because it becomes difficult to code nonspecific terms, but this has not been studied.

If there is great uncertainty on how adverse events are coded it will lead to non-differential misclassification. This will underestimate the relationship and may result in failure to detect important adverse events.

With MedDRA it is possible to match the investigator's verbatim descriptions more closely because of the increasing amount of terms. The drawback is that it becomes harder to statistically detect adverse events that are related but do not present themselves in the same way in each patient, i.e. signal dilution, because events are split into subcategories. Advanced searching and data analysis in MedDRA, where related categories and Preferred Terms are lumped together, have been developed to try and counteract this problem, but a survey showed that that these tools are not used [15]. The recommendations by the expert group on when to explore adverse events are vague and it is even recommended to design the analysis post hoc [16], which carries a risk of bias. If these problems could be solved, it would lead to more transparent handling of adverse events. Better guidelines would need to be developed by regulatory authorities.

Because of “background noise”, summary tables at SOC level are usually only efficient in finding adverse events that occur frequently in the treatment group and rarely in the placebo group. For example, if a trial runs over 2 years, most patients in both the treatment and the placebo group might have experienced headaches. With such background noise, it will be almost impossible to detect any other neurological diseases or symptoms at the SOC level.

Another problem with summary tables at SOC level is that sometimes related adverse events are not even in the same category. In MedDRA there are disorders that are defined by laboratory tests . For example, “Hepatic function abnormal” belongs to the SOC “Hepatobiliary disorders” whereas “liver function test abnormal” belongs to “Investigations”. In a summary table, these identical adverse events would be presented in two different categories.

The CONSORT group recommends that coding strategies should be reported [18]. They also recommend that adverse events should be defined. Unfortunately, MedDRA doesn't hold any formal definitions of adverse events. In the protocol, one can of course define important expected adverse events, but the consequence is that the investigator and the coder will have to look in two different systems. The usage of definitions is usually limited. Lack of definitions is an important limitation and makes comparison of harms between different trials problematic.

The many ways to define treatment emergent adverse events, and hence censor adverse events, can result in bias because data analysis is often done unblinded [19] and the most favourable strategy might be chosen.

In package inserts, common and serious adverse events are reported. With MedDRA, we get a greater variety of adverse events but each one becomes less frequent. As the package inserts are mainly based on frequency, we would expect the total number of adverse events to go down using MedDRA. This makes it difficult to compare adverse events historically and newer package inserts should therefore be interpreted cautiously.

The increased specificity of MedDRA terms might be partly responsible for the common failure to detect important adverse events before drug approval, leading to many patients being harmed by dangerous drugs. In post-marketing surveillance studies, sensitive techniques for detecting adverse events have been developed, e.g. data mining and lumping of hundreds of related terms to counteract the problems of splitting adverse events. However, as observational studies can only detect strong signals reliably, we should have more emphasis on detecting adverse events in the clinical trials, perhaps by using some of the same techniques.

The organization behind MedDRA advertises that it is clinically validated but defines this as “developed and maintained by medical experts” [4]. This is not a guarantee that coding in MedDRA is reproducible nor is it a guarantee that adverse events are identified as well as, or better than, previous dictionaries. It is essential that the many different ways to define and handle adverse events becomes standardized or at least documented. To decrease interobserver variation coders and investigators should be meticulous and well trained. We recommend that a thorough interobserver study of coding should be performed elucidating both the magnitude and the nature of the problems with variability in coding. Brown 1997 [10] investigated differences in the accuracy of coding and incidence of adverse events in a clinical trial of an unspecified neuroleptic drug using COSTART and MedDRA. Since MedDRA has changed significantly over the past 15 years, the ability of MedDRA to identify known adverse events compared with older dictionaries should be re-evaluated using trial data for a known drug.

### Limitations

Because of the constant development of MedDRA, the results of the interobserver study and other studies we have included might no longer apply. Our study might be subject to publication bias since MedDRA is predominately used by the pharmaceutical industry, which might have experimented with MedDRA during its implementation without publishing their results.

### Conclusion

Important differences in coding between two coders exist but the consequences have been poorly elucidated. The implementation of MedDRA has led to a more specific coding system but it has made signal detection much more difficult and has had great consequences for product labeling. Strategies to improve detection of adverse events have been developed but are rarely used. It is very surprising that so little research has been performed in this important area for public health. This needs to be remedied.

## Supporting Information

Checklist S1 **PRISMA checklist.**
(DOC)

Text S1 **Search strategy for PubMed, Cochrane and EMBASE.**
(DOCX)
