# Supplementary material for: Challenges in Coding Adverse Events in Clinical Trials: A Systematic Review
Source: PLoS One. 2012 Jul 20;7(7):e41174. doi: 10.1371/journal.pone.0041174 (PMC3401103; doi:10.1371/journal.pone.0041174)
Supplement: Text S1 — Search strategy for PubMed, Cochrane and EMBASE. (DOCX) [file pone.0041174.s002.docx]

Search strategy (PubMed and Cochrane):

| 1. Meddra 2. snomed and (adverse or ae or aer or adr or adrs or side or ade or ades or "drug reaction" or "drug reactions") 3. who-art 4. "medical coding" 5. ctcae and (interobserver* or intraobserver* or kappa* or interrater* or intrarater* or interclass or intraclass) 6. costart 7. "preferred term" and (adverse or ae or aer or adr or adrs or side or ade or ades or "drug reaction" or "drug reactions") 8. "Adverse Drug Reaction Reporting Systems"[MAJR] and (interobserver* or intraobserver* or kappa* or interrater* or intrarater* or interclass or intraclass) 9. (adverse or ae or aer or adr or adrs or side or ade or ades or "drug reaction" or "drug reactions") and (code* or coding) and (interobserver* or intraobserver* or kappa* or interrater* or intrarater* or interclass or intraclass) 10. 1 or 2 or 3 or 4 or 5 or 6 or 7 or 8 or 9 |
| --- |

Search strategy (EMBASE):

| 1. meddra.ti or (meddra and (code* or coding) and (adverse or ae? or adr? or side or ade? or drug reaction?) ) 2. snomed and (adverse or ae? or adr? or side or ade? or drug reaction?) 3. who-art and (adverse or ae? or adr? or side or ade? or drug reaction?) 4. medical coding 5. ctcae and (interobserver? or intraobserver? or kappa? or interrater? or intrarater? or interclass or intraclass or observers) 6. costart 7. "preferred term" and (adverse or ae? or adr? or side or ade? or drug reaction?) 8. *adverse drug reaction/ and (interobserver? or intraobserver? or kappa? or interrater? or intrarater? or interclass or intraclass) and (code* or coding or identif* or categor* or validat* or classif*) 9. (adverse or ae? or adr? or side or ade? or drug reaction?) and (coding or code*) and (interobserver? or intraobserver? or kappa? or interrater? or intrarater? or interclass or intraclass) 10. 1 or 2 or 3 or 4 or 5 or 6 or 7 or 8 or 9 |
| --- |
